# *EEPD1* attenuates radiation-induced cardiac hypertrophy and apoptosis by degrading *FOXO3A* in cardiomyocytes: Effect of *EEPD1* on radiation induced cardiomyopathy

**DOI:** 10.3724/abbs.2024130

**Published:** 2024-08-29

**Authors:** Kaiwen Yu, Xi Su, Tongfang Zhou, Xuwei Cai, Min Zhang

**Affiliations:** 1 Department of Cardiology Shanghai Jiao Tong University Affiliated Chest Hospital Shanghai 200030 China; 2 Radiotherapy Department of Shanghai Jiao Tong University Affiliated Chest Hospital Shanghai 200030 China

**Keywords:** *EEPD1*, radiation-induced heart disease, cardiac hypertrophy, apoptosis, *FOXO3A*

## Abstract

Radiation-induced heart disease (RIHD) is a severe delayed complication of thoracic irradiation (IR). Endonuclease/exonuclease/phosphatase family domain-containing 1 ( *EEPD1*) plays an important role in DNA damage repair, but its role in RIHD is less known. In this study, *EEPD1* global knockout mice, C57BL/6J mice, and C57BL/6J mice overexpressing *EEPD1* are treated with radiation at a total dose of 20 Gy or 0 Gy. After 9 weeks, echocardiography is used to assess cardiac hypertrophy and apoptosis. The results show that *EEPD1* deletion exacerbates radiation-induced cardiac hypertrophy and apoptosis, while *EEPD1* overexpression has the opposite effect. Further mechanistic investigations reveal that *EEPD1* interacts with *FOXO3A* and destabilizes it by catalyzing its deubiquitination. Inhibition of *FOXO3A* ameliorates cardiac hypertrophy and apoptosis after *EEPD1* knockdown. Thus, *EEPD1* protects against radiation-induced cardiac hypertrophy and apoptosis via destabilization of *FOXO3A*, which may offer new insight into therapeutic strategies for RIHD.

## Introduction

Radiation-induced heart disease (RIHD) is a significant adverse effect of radiation therapy in the treatment of thoracic tumors, breast cancer, chest wall malignancies, and lymphoma [1]. The pathologies of RIHD are chronic progressive processes. Previous studies have shown that endothelial cell damage, especially in microvascular cells, may play an important role in RIHD [2]. Oxidative stress, the DNA damage response, telomere erosion, and mitochondrial dysfunction are also important causes of RIHD [ 3, 4]. Despite these findings, the molecular mechanisms underlying RIHD remain incompletely understood.

In 2015, the up-regulation of endonuclease/exonuclease/phosphatase family domain-containing 1 ( *EEPD1*) was detected in embryonic stem cells following DNA damage [5]. Moreover, several studies have revealed that *EEPD1* plays a crucial role in the pathology of many diseases, including esophageal squamous cell carcinoma [6], acute myeloid leukemia [7] and breast cancer [8]. Because many of these diseases tend to occur in the thoracic region, we speculated that *EEPD1* expression may be correlated with RIHD.

The *EEPD1* gene belongs to the ribonuclease gene family. It has been demonstrated that *EEPD1* is recruited to stalled replication forks during replication stress, where it promotes their restart. Our study demonstrated the important role of *EEPD1* in regulating cardiac apoptosis and hypertrophic RIHD.

A member of the FOXO subfamily, *FOXO3A*, was first identified in the human placental cosmid. It mediates a variety of cellular processes, including apoptosis, proliferation, cell cycle progression and DNA damage. It also responds to several cellular stresses, such as UV irradiation and oxidative stress. There is a close relationship between *FOXO3A* and cardiovascular diseases such as cardiac hypertrophy [ 9, 10], cardiac ischemia/reperfusion [11], and atherosclerosis [12]. However, the role of *FOXO3A* in RIHD has not been explored.

In this study, we found that *EEPD1* expression decreases in RIHD, which enhances *FOXO3A* level and exacerbates cardiac hypertrophy and apoptosis. These effects are ameliorated by *FOXO3A* inhibition. In conclusion, the *EEPD1*- *FOXO3A* axis plays a significant role in RIHD.

## Materials and Methods

### Animal study

The *EEPD1*-knockout (EKO) mouse model was established by the Shanghai Nanfang Research Center for Model Organisms (Shanghai, China) using the CRISPR-Cas9 system. During this process, four guide RNAs (gRNA1, 5′-ctctatccccagagatccct-3′; gRNA2, 5′-acatcctggtgaatcaggag-3′; gRNA3, 5′-tggtgtaggggccaccaaac-3′; and gRNA4, 5′-agcactctcccagttccctg-3′) were used to cleave the whole-genome sequence of *EEPD1*. The following genotyping primers were used for the EKO mice: *EEPD1* genotyping forward outer primer 5′-cttcctcgacccttaagtcctgta-3′, *EEPD1* genotyping reverse outer primer 5′-atgctgagggccatcttctcg-3′, *EEPD1* genotyping forward inner primer 5′-taacctcccgttcttttgtgcct-3′, and *EEPD1* genotyping reverse inner primer 5′-ccggacactcatgagctgag-3′. All animal experiment procedures were reviewed and approved by the Shanghai Jiao Tong University Animal Care Committee. All experiments and measurements were carried out in a blinded manner. For whole heart irradiation, the mice were subjected to cardiac irradiation with a small-animal radiation research platform (SARRP; XStrahl Medical and Life Sciences, Surrey, UK) at Shanghai Chest Hospital. The procedures were performed as described previously [13]. Briefly, all of the mice were fixed on a board after being anaesthetized with intraperitoneal injection of 10% chloral hydrate. The contours of the whole heart were delineated and examined in CT images. We localized the central point of the heart and then fixed the mice on a small-animal radiation research platform (SARRP). Radiation was delivered at 220 kV at 13 mA for a total dose of 20 or 0 Gy/min [ 14– 16]. All animals were housed under a 12 h/12 h light/dark cycle (lights on 8:00–20:00) at 23°C and 40%–70% humidity. *Foxo3A* knockdown vector and an empty vector as a negative control were constructed by ObiO (Shanghai, China). Eight-week-old WT and KO mice were injected with 200 μL of virus containing 1×10 ^12^ vector genomes via the tail vein. After four weeks, the mice were subjected to radiotherapy treatment.

### Echocardiography

Mice were anaesthetized by intraperitoneal injection of 4% chloral hydrate (0.1 mL/10 g body weight) and examined by M-mode echocardiography using a Vevo2100 Imaging System (VisualSonics, Toronto, Canada) at 8 weeks after radiotherapy. M-mode tracings of the left ventricle (LV) were acquired using the short-axis view, with the ultrasound beam perpendicular to the LV at the midpapillary level to determine the EF, FS, wall thickness, LV inner diameter, and LV volume.

### LDH and CK-MB release in serum

The blood samples were centrifuged at 3000 *g* for 15 min. Then, the plasma samples were stored at −80°C for subsequent analyses. Lactate dehydrogenase (LDH) and creatine kinase-MB (CK-MB) levels were determined using the corresponding kits (C018-a) provided by Changchun Huili Biotech (Changchun, China) according to the manufacturer’s instructions.

### Masson staining

The completed heart paraffin sections were deparaffinized in water through a series of steps: xylene I, II, and III for 5 min each, followed by immersion in absolute ethanol for 1 min, 95% ethanol for 1 min, 85% ethanol for 1 min, and 75% ethanol for 1 min. The sections were briefly rinsed in tap water and then stained with hematoxylin for 5 min or as necessary. Subsequently, staining was performed with Weigert’s iron hematoxylin for 5–10 min, followed by rinsing under running water. Differentiation was carried out in 1% hydrochloric acid alcohol, with a brief rinse under running water. The sections were then stained with Mallory’s acid fuchsin solution for 5‒10 min, followed by a slight rinse with distilled water. After treatment with a 1% phosphomolybdic acid aqueous solution for approximately 5 min, the sections were counterstained directly with aniline blue or green solution for 5 min. A brief treatment with 1% glacial acetic acid for 1 min was performed before dehydration in 95% ethanol several times. Finally, the sections were cleared in absolute ethanol, cleared in xylene, and mounted with neutral mounting medium.

### Sirius Red staining

The completed heart paraffin sections were deparaffinized in water through a series of steps: xylene I, II, and III for 5 min each, absolute ethanol for 1 minute, 95% ethanol for 1 min, 85% ethanol for 1 min, and 75% ethanol for 1 min. The sections were briefly rinsed in tap water and then stained with hematoxylin for 5 min or as necessary. Next, the sections were stained with Weigert’s iron hematoxylin staining solution for 5‒10 min, followed by rinsing in distilled water for 10‒20 s to remove excess staining solution. Subsequently, the sections were rinsed in tap water for 5 min. Sirius Red staining solution was added to the sections, which were then allowed to stain for 15‒30 min. The sections were then gently rinsed under running water to remove excess staining solution from the surface of the slides. Dehydration and clearing were performed as follows: 75% ethanol for 1 min, 95% ethanol for 1 min, and absolute ethanol for 1 min, followed by three changes of xylene, each for 1‒2 min. Finally, the sections were mounted with neutral mounting medium for permanent fixation.

### WGA staining

The completed heart paraffin sections were initially deparaffinized in water, successively treated with xylene I, II, and III for 5 min each, followed by immersion in absolute ethanol for 1 min, 95% ethanol for 1 min, 85% ethanol for 1 min, and 75% ethanol for 1 min, and then briefly rinsed in tap water. Subsequently, the sections were washed three times with PBS for 5 min each. Afterward, they were subjected to a 10-min incubation in 0.1% Triton X-100 (in PBS), followed by three additional PBS washes of 5 min each. The sections were then stained with a solution containing 15 μg/mL WGA for 60 min, followed by another three PBS washes of 5 min each. Further staining was performed by incubating the sections with DAPI staining solution for 10 min, followed by three final PBS washes of 5 min each. Finally, the slides were inverted, anti-fading mounting medium was applied, and fluorescence was observed.

### Cell culture and treatment

HEK293T cells (SCSP-502) were purchased from the Cell Bank of the Chinese Academy of Sciences (Shanghai, China). HEK293T cells were maintained in DMEM supplemented with high glucose (4.5 g/L; 11965092; Gibco, Carlsbad, USA), L-glutamine, 10% fetal bovine serum (10099158; Gibco) and 1% penicillin/streptomycin (15140122; Gibco). HEK293T cells were cultured in 10-cm dishes until 60%–70% confluency and transiently transfected with 4 μg of each plasmid DNA for 48 h using Lipofectamine 3000 transfection reagent (L3000015; Invitrogen, Carlsbad, USA) following the manufacturer’s protocols.

Primary cardiomyocytes were obtained from 1-day-old rat pups as previously described [2]. Neonatal rat cardiomyocytes (NRCMs) and cardiac fibroblasts (NRCFs) were separated by distinct adhesion time. NRCMs were then resuspended in DMEM supplemented with 10% FBS and seeded at a density of 2×10 ^5^ cells/mL in culture dishes or flasks [17]. Gene overexpression or knockdown was performed by adeno-associated virus (AAV) infection at a multiplicity of infection of 60 plaque forming unit followed by treatment with radiation at 0 or 16 Gy. The sequences of the shRNAs were as follows: sh- *EEPD1*, 5′-CGAAGUCUCUGGACAACAU-3′ and sh-NC 5′-UUCUCCGAACGUGUCACGU-3′.

### Plasmids

To induce the expression of exogenous EEPD1 and its various domain deletions, we employed pCDH-CMV-MCS-EF1-RFP-T2A-Puro vector (ViGene Biosciences, Jinan, China). EEPD1 and its various domain deletions complementary DNA (cDNA) was first amplified by PCR from pCDH-CMV-MCS-EF1 plasmid, and a 3× Flag-tag was added at the C terminus of EEPD1. The PCR products were digested with *Eco*RI and *Not*I and inserted into *Eco* RI/ *Not*I-digested to RFP-T2A-Puro vector construct targeted vector.

### Western blot analysis

Cells or heart tissue were collected and homogenized in lysis buffer (50 mM Tris, 150 mM NaCl, 1% NP-40, and 1% sodium deoxycholate) containing proteinase and phosphatase inhibitors (Roche, Basel, Switzerland) for protein extraction. After denaturation, 30 μg of protein was subject to SDS-PAGE, followed by transfer onto PVDF membranes. After being blocked with 5% BSA in TBST (50 mM 30 Tris-HCl, 150 mM NaCl, and 0.2% Tween-20) for 1 h at room temperature, the samples on the PVDF membrane were incubated overnight at 4°C with the indicated primary antibodies diluted at 1:1000 in blocking buffer. After 3 times of wash and the membranes were incubated with HRP-conjugated anti-IgG antibodies (1:5000; Jackson, West Grove, USA) for 1 h. Chemiluminescence detection was performed using enhanced chemiluminescence (ECL) reagent on an Amersham Imager 680 (Amersham, Buckinghamshire, UK).

The primary antibodies used in this study were as follows: anti-Bcl2 (1:1000, 3498; CST, Beverly, USA), anti-Bax (1:1000, 14796; CST), anti-GAPDH (1:1000, 5174; CST), Myh7 (1:1000, sc-53089; Santa Cruz, Santa Cruz, USA), anti-TGF Beta1 (1:1000, 21898-1-AP; Proteintech, Wuhan, China), anti-BNP (1:1000, PA5-96084; Invitrogen), anti-Collagen I (1:1000, ab270993; Abcam, Cambridge, UK), anti-FOXO3A (1:1000, 66428-1-Ig; Proteintech), and anti-EEPD1 (1:1000, 24310-1-AP; Proteintech).

### Immunoprecipitation assay

HEK293T cells were lysed in Pierce IP lysis buffer (87788; Thermo Fisher Scientific, Waltham, USA) supplemented with phosphatase inhibitor cocktail (04906837001; Roche), protease inhibitor cocktail (05892970001; Roche) and NEM (E3876; Sigma, St Louis, USA) on ice for 30 min. Lysate was subject to centrifugation at 13,000 *g* for 15 min at 4°C to obtain the cell extracts. The cell extracts were incubated with anti-HA Co-IP beads (SB-PR003; Share-Bio, Shanghai, China) or anti-Flag Co-IP beads (SB-PR002; Share-Bio) at 4°C for 12 h, after which the pellets were washed three times with PBST (phosphate-buffered saline with Tween-20). The bound proteins were eluted by boiling the beads in 2× sample buffer, followed by western blot analysis.

### Protein stability experiment

HEK293T cells were transfected with the HA- *FOXO3A* or HA- *FOXO3A* and Flag-EEPD1, and then treated with 10 μM CHX (HY-12320; MCE, New Jersey, US) for the indicated time periods. After collection of proteins, Western blot analysis was performed.

### Immunofluorescence staining

NRCMs were seeded onto laminin-coated coverslips prior to infection with ShControl or Sh *EEPD1* for 24 h, followed by radiation therapy (0 or 16 Gy). The cells were washed with PBS and postfixed in 4% paraformaldehyde for 15 min at 20°C‒23°C. After being blocked in PBS containing 5% goat serum albumin and 0.2% Triton X-100 for 10 min, the cells were incubated overnight at 4°C with anti-EEPD1 antibody (1:200, HPA053668; Sigma) and anti-FOXO3A antibody (1:200, 66428-1-Ig; Proteintech). After wash with PBS three times for 5 min, the cells were incubated with goat anti-rabbit IgG (H+L) highly cross-adsorbed with Alexa Fluor 488 (1:500, A-21429; Invitrogen) and goat anti-mouse IgG (H+L) highly cross-adsorbed with Alexa Fluor 555 (1:500, A-32723; Invitrogen) for 1 h at 20‒23°C, and nuclear staining was performed with 4′,6-diamidino-2-phenylindole (DAPI; 100 ng/mL) for 5 min. In terms of cell area measurement, NRCMs were incubated overnight at 4°C with anti-EEPD1 antibody (1:200, HPA053668; Sigma) and anti-Actinin α2 antibody (1:200, A7811; Sigma). After wash with PBS three times for 5 min, the cells were incubated with goat anti-rabbit IgG (H+L) highly cross-adsorbed with Alexa Fluor 488 (1:500, A-21429; Invitrogen) and goat anti-mouse IgG (H+L) highly cross-adsorbed with Alexa Fluor 555 (1:500, A-32723; Invitrogen) for 1 h at 20‒23°C, and nuclear staining was performed with 4′,6-diamidino-2-phenylindole (DAPI; 100 ng/mL) for 5 min. Finally, cell images were captured under a fluorescence microscope.

### Apoptosis assay

The hearts of wild-type (WT) NC, KO NC, WT radiotherapy (RT), KO RT, KO RT ShFoxo3a, KO RT, WT RT ShFoxo3a and WT RT mice were harvested and cross-sectioned to prepare paraffin sections. Apoptosis was determined using a TdT-mediated dUTP Nick-End Labeling (TUNEL) assay kit (G1501; Servicebio, Shanghai, China). The nuclei were stained with DAPI (G1012; Servicebio). Immunofluorescence was analyzed under a fluorescence microscope (Carl Zeiss, Wetzlar, Germany).

### Immunohistochemistry

For immunohistochemical staining, the sections were deparaffinized in xylene and rehydrated. Antigen retrieval was performed with protease K at 37°C for 15 min. We used 3% H _2_O _2_ to block the activity of endogenous peroxidase. The sections were then incubated overnight at 4°C with anti-EEPD1 antibody (1:200, sc-398028; Santa Cruz). After three washes in PBS, biotinylated secondary antibodies were added and incubated for 1 h at room temperature, followed by color development with a DAB kit (ZSGB-Bio, Beijing, China). The sections were examined using a microscope (ECLIPSE Ci-S; Nikon, Tokyo, Japan).

### Proximity ligation assay

We performed an *in situ* analysis of the interaction between EEPD1 and FOXO3A using a proximity ligation assay (PLA) kit (Sigma). The experiment was performed in accordance with the manufacturer’s instructions. Briefly, NRCMs were fixed and permeabilized according to the instructions for immunofluorescence microscopy. Following FOXO3A (1:100, sc-48348; Santa Cruz) and EEPD1 (1:100, HPA053668; Sigma) antibody incubation, the slides were washed with PBS and incubated with secondary antibodies conjugated to oligonucleotides (DUO82002 and DUO82004; Sigma) for 1 h at 37°C. After probe incubation, the samples were incubated in ligation solution for 1 h at 37°C. For 100 min at 37°C, rolling circle amplification was performed on the generated DNA circle, followed by hybridization with fluorescently labelled nucleotides. After DAPI staining, the sections were observed with a confocal microscope (LSM900; Carl Zeiss).

### Flow cytometry

To compare apoptosis levels between the groups, fluorescence intensity values were normalized to those of the control group. An Annexin V-APC/7-AAD apoptosis detection kit (AT105; Multi Sciences, Hangzhou, China) was used according to the manufacturer’s instructions. NRCMs were divided into four groups: ShControl, ShEEPD1, ShControl+RT, and ShEEPD1+RT. After infecting with the adeno-associated virus for 24 h, the cells were subjected to 0 or 16 Gy of radiotherapy treatment, respectively. The cells were then collected for flow cytometry analysis 48 h post-treatment.

### Statistical analysis

All experiments were carried out with at least three biological replicates. Data are presented as the mean ± standard deviation (SD). Statistical differences were determined by one-way ANOVA or Student’s *t* test using GraphPad Prism 8 (GraphPad Software, San Diego, USA). *P*<0.05 was considered statistically significant.

## Results

### Downregulation of EEPD1 in a mouse RIHD model

**Figure 1:**
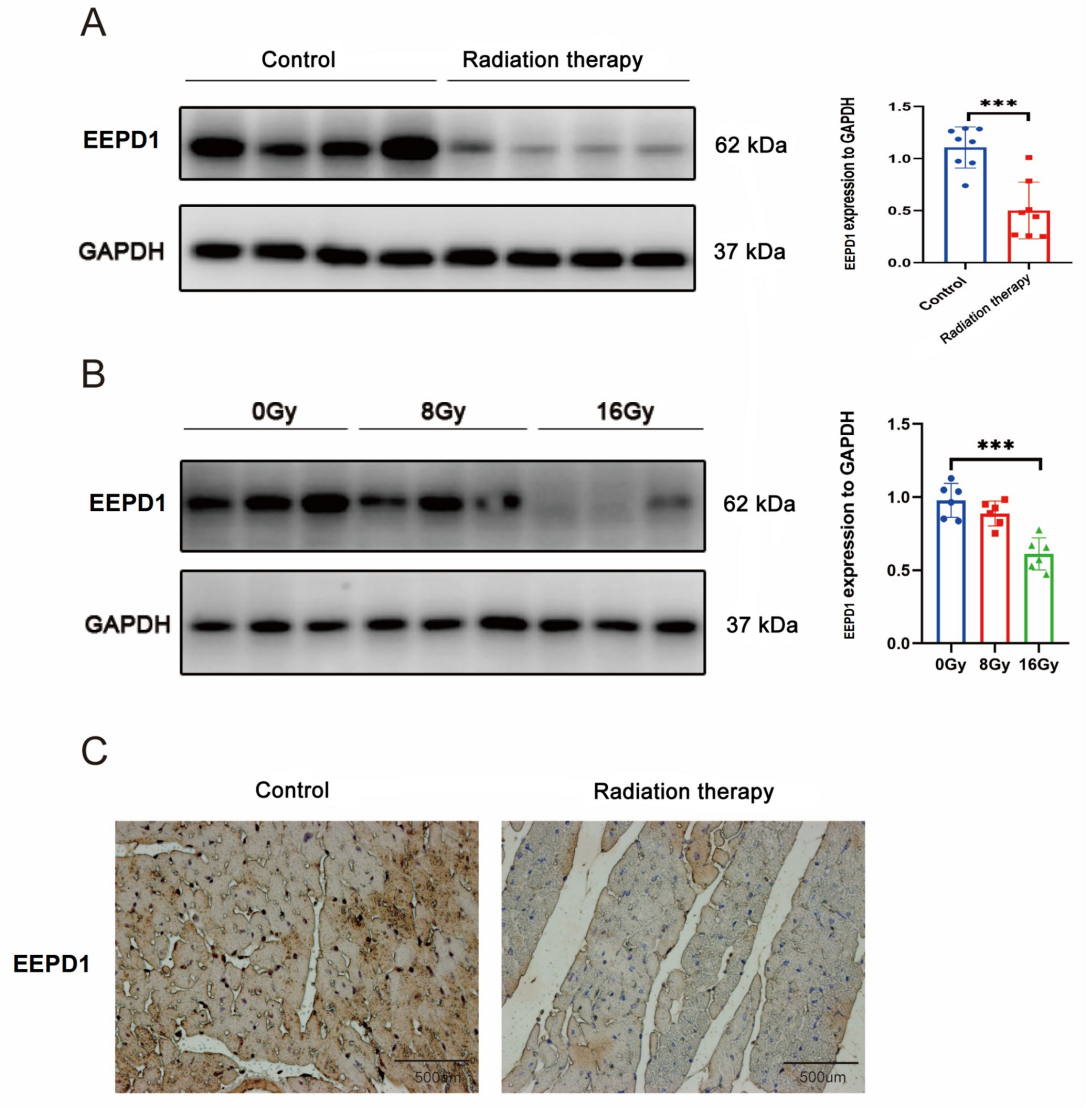
Figure 1 Downregulation of *EEPD1* in a mouse RIHD model (A) Representative in vivo immunoblot images. n=8 for each group. (B) Representative in vitro immunoblot images. n=6 for each group. (C) Representative immunohistochemical (IHC) staining of the EEPD1 protein. Scale bar: 500 μm. ***P<0.001.

To evaluate the role of *EEPD1* in RIHD, we first examined changes in its expression after radiotherapy *in vivo*. Western blot analysis and immunohistochemistry revealed that the protein level of EEPD1 significantly decreased after 20 Gy of radiotherapy ( Figure 1A,C). Consistent with our *in vivo* results, the EEPD1 protein level obviously decreased after 16 Gy of radiotherapy ( Figure 1B). We demonstrated the success of the model by Masson staining, WGA staining, echocardiography, cardiac enzyme analysis, and an altered survival rate ( Supplementary Figure S1). These findings suggested that EEPD1 may be involved in RIHD.

### *EEPD1* deletion exacerbates radiation-induced cardiac hypertrophy

**Figure 2:**
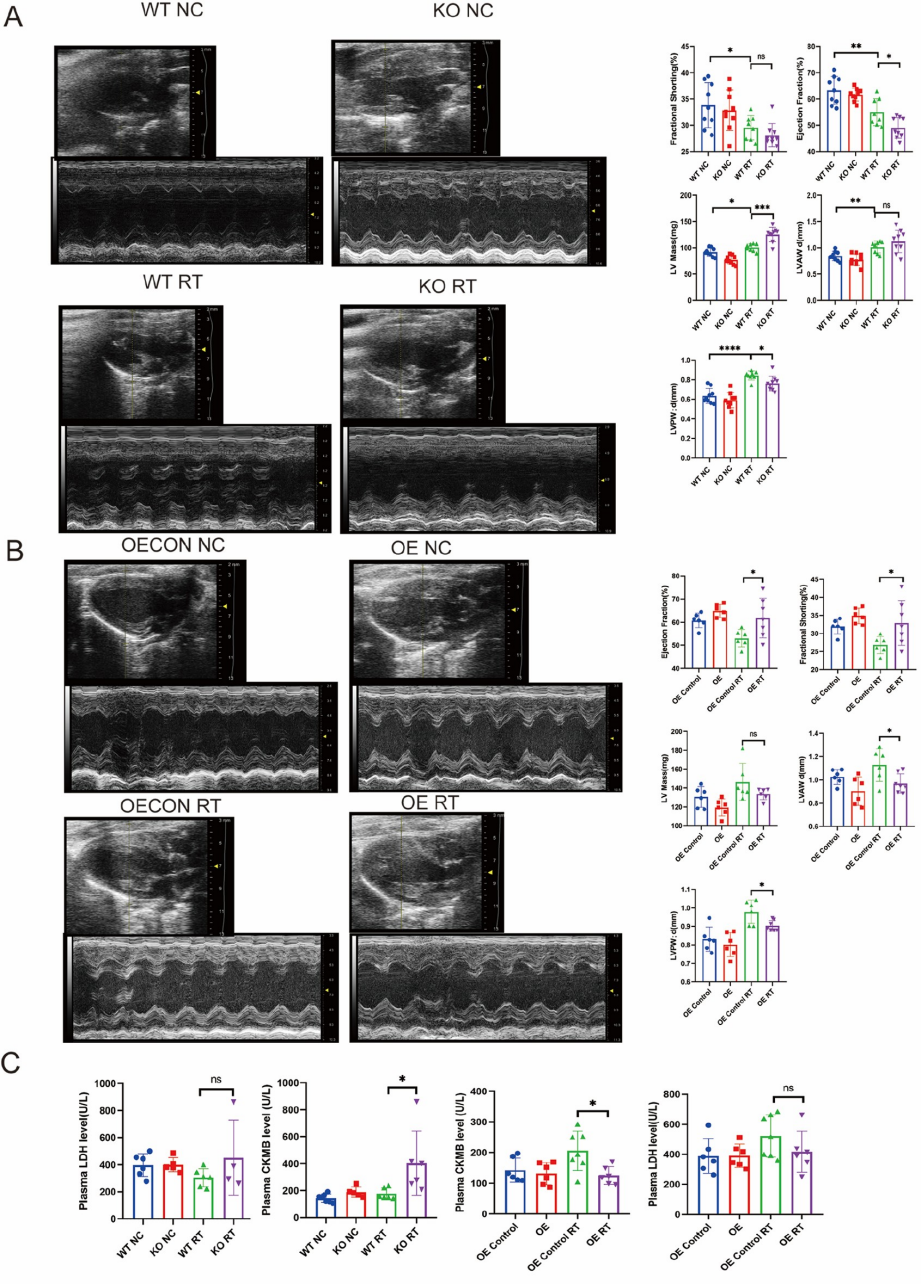
Figure 2 *EEPD1* deletion or overexpression regulates cardiac function after radiotherapy (A,B) Representative images of echocardiographs and statistics of the ejection fraction (EF), fractional shortening (FS), LV mass, LVAW d and LVPW d. (C) Effect of EEPD1 deficiency and EEPD1 overexpression on CKMB and LDH release after radiotherapy. *P<0.05, **P<0.01, ***P<0.001, ****P<0.0001.

**Figure 3:**
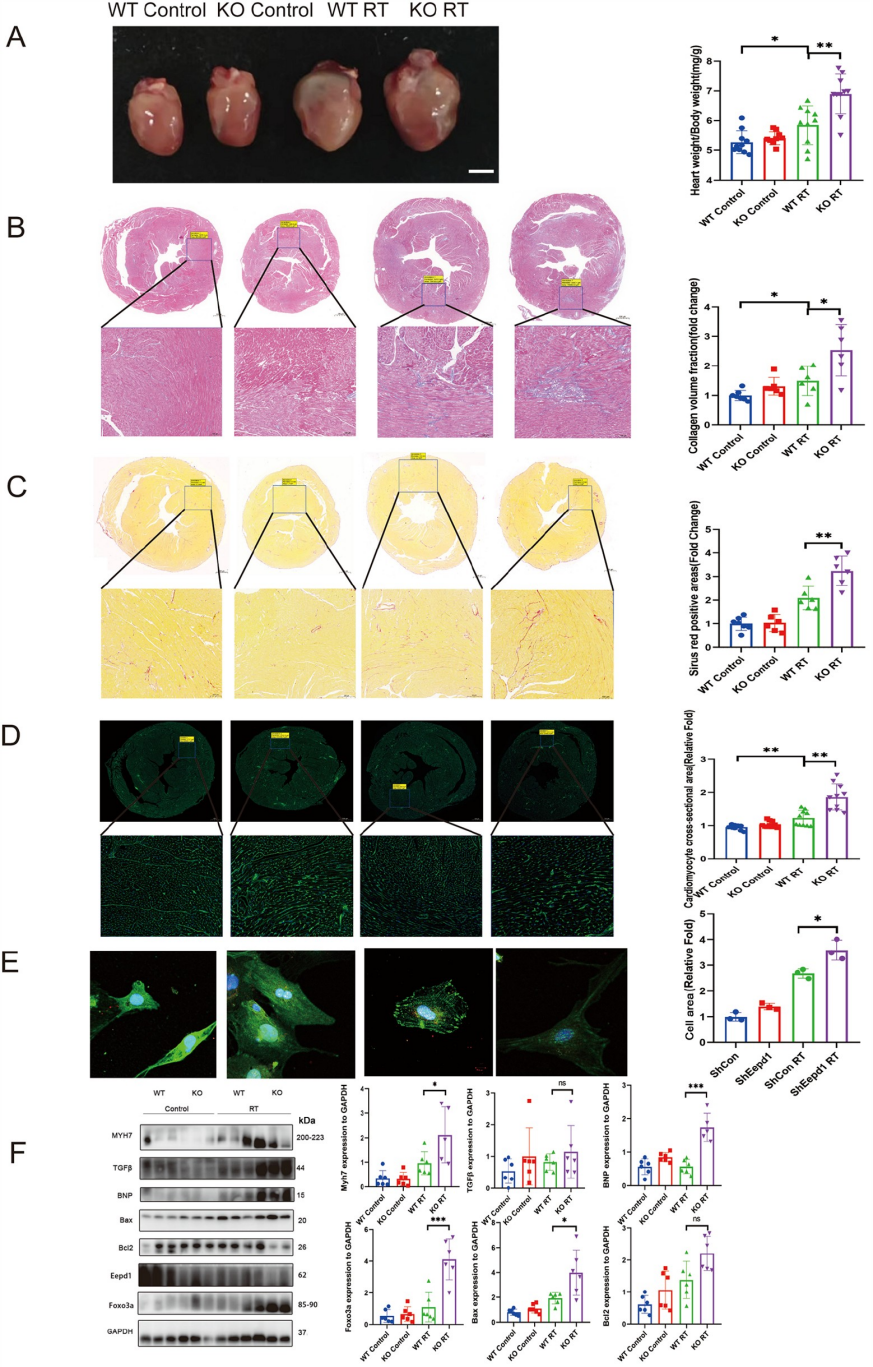
Figure 3 *EEPD1* deletion exacerbates radiation-induced cardiac hypertrophy (A) Representative whole heart images. Scale bar: 2.5 mm. Myocardial fibrosis was detected by Masson’s trichrome staining. Scale bar: 500 μm (upper) and 100 μm (lower). (C) Myocardial fibrosis detected by Sirius Red staining. Scale bar: 500 μm (upper) and 100 μm (lower). (D) WGA staining showing myocyte hypertrophy. Scale bar: 500 μm (upper), 100 μm (WT Control, KO RT lower), and 200 μm (KO Control, WT RT lower). (E) Immunofluorescence of NRCMs and related quantification. Scale bar: 10 μm. n=3. (F) Representative western blots and related quantification. *P<0.05, **P<0.01, ***P<0.001.

To further investigate the role of *EEPD1* in radiation-induced cardiac hypertrophy *in vivo*, we constructed *EEPD1*-knockout mice via the CRISPR-Cas9 system ( Supplementary Figure S2). Echocardiographic analysis revealed that 2 months after radiotherapy, although there was a trend toward lower fraction shortening in the KO group than in the wild-type (WT) group, the difference did not reach statistical significance. Notably, the left ventricular mass (LV mass) significantly increased. The ejection fraction and left ventricular posterior wall at end-diastole (LVPW; d) were significantly lower in the KO group than in the WT group ( Figure 2A). As predicted, the WT mice exhibited marked cardiac hypertrophy after radiation, as evidenced by increased size, increased heart weight/body weight ratios, and large areas of fibrosis ( Figure 3A–D). Furthermore, gross morphology of the heart, Masson’s trichrome staining, Sirius Red staining and WGA staining revealed more severe cardiac fibrosis and hypertrophy in the KO mice than in the WT mice after radiotherapy. However, no significant differences were observed in these features between the WT and KO groups without radiotherapy ( Figure 3A–C). The protein levels of Myh7 and BNP were significantly greater in the KO mice than in the WT mice after radiotherapy. There was a trend toward greater TGFβ protein level in the KO group than in the WT group, but the difference did not reach statistical significance. A slight upwards trend in BNP and TGFβ was observed in the KO group compared with those in the WT group without radiotherapy, and no difference in MYH7 was observed between the WT and KO groups without radiotherapy ( Figure 3F). Consistent with our *in vivo* results, analysis of the cell area using α-actinin staining revealed that Sh *EEPD1* significantly increased the cell area of NRCMs after radiotherapy compared to that in the control group ( Figure 3E). Together, these results showed that *EEPD1* deficiency exacerbates radiation-induced cardiac hypertrophy.

### *EEPD1* deletion exacerbates radiation-induced cardiomyocyte apoptosis

**Figure 4:**
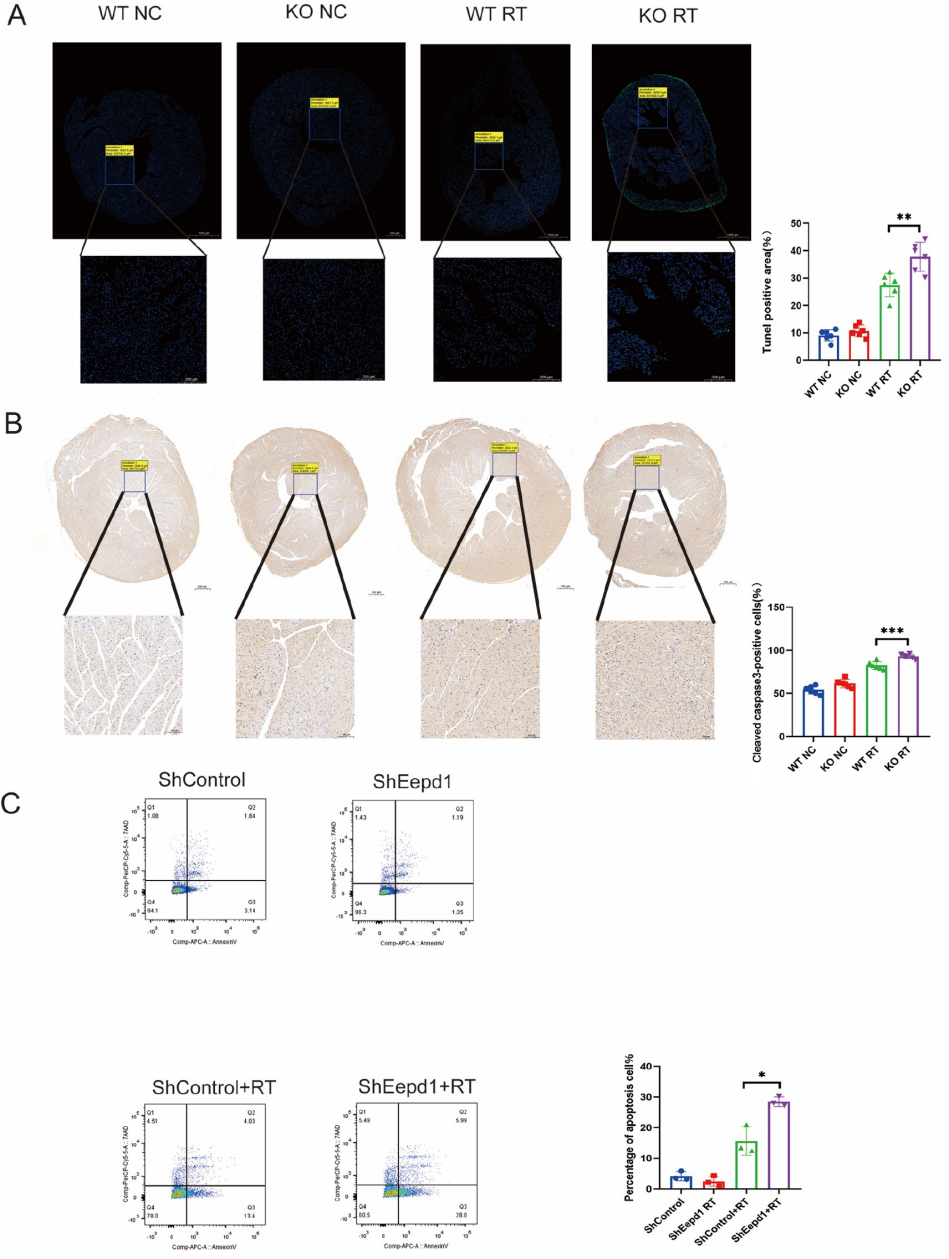
Figure 4 *EEPD1* deletion exacerbates radiation-induced cardiomyocyte apoptosis (A) TUNEL staining of each group (n=6). Scale bar: 500 μm (upper) and 100 μm (lower). (B) Representative immunohistochemical (IHC) staining of cleaved caspase-3 protein (n=6). Scale bar: 500 μm (upper) and 100 μm (lower). (C) Flow cytometry results with Annexin V-APC/7AAD staining. n=3. *P<0.05, **P<0.01, *** P<0.001.

Cardiomyocyte apoptosis has been implicated as one of the mechanisms underlying radiation-induced cardiomyopathy. After the compensatory phase of cardiac hypertrophy, cardiomyocyte apoptosis typically follows. As shown in Figure 4A,B, both the TUNEL-positive area and cleaved caspase3-positive area were significantly greater in *EEPD1*-deficient mice than in WT mice after radiotherapy. A slight increase in the TUNEL-positive area and cleaved caspase3-positive area was observed in the KO group compared with the WT group without radiotherapy. It is well known that cells that are positive for both TUNEL and cleaved caspase3 are apoptotic cells, while cells that are negative for cleaved caspase3 but positive for TUNEL are known as necrotic cells. Bax protein level was significantly greater in *EEPD1* knockout mice than in WT mice after radiotherapy ( Figure 3F). There was a trend toward lower Bcl2 protein level in the KO group than in the WT group, but the difference did not reach statistical significance ( Figure 3F). A slight upwards trend in Bax and Bcl2 expressions was observed in the KO group compared to those in the WT group in the absence of radiotherapy ( Figure 3F). Following radiation, plasma CK-MB level was significantly greater in KO mice than in WT mice, while plasma LDH level was not significantly different between the two groups ( Figure 2C). Furthermore, flow cytometry analysis of NRCMs with Annexin V-APC/7AAD staining revealed that *EEPD1* shRNA infection significantly exacerbated apoptosis after radiotherapy, with no discernible difference in apoptosis between the *EEPD1* shRNA and control shRNA groups in the absence of radiotherapy ( Figure 4C).

### *EEPD1* overexpression ameliorates radiation-induced cardiac hypertrophy

**Figure 5:**
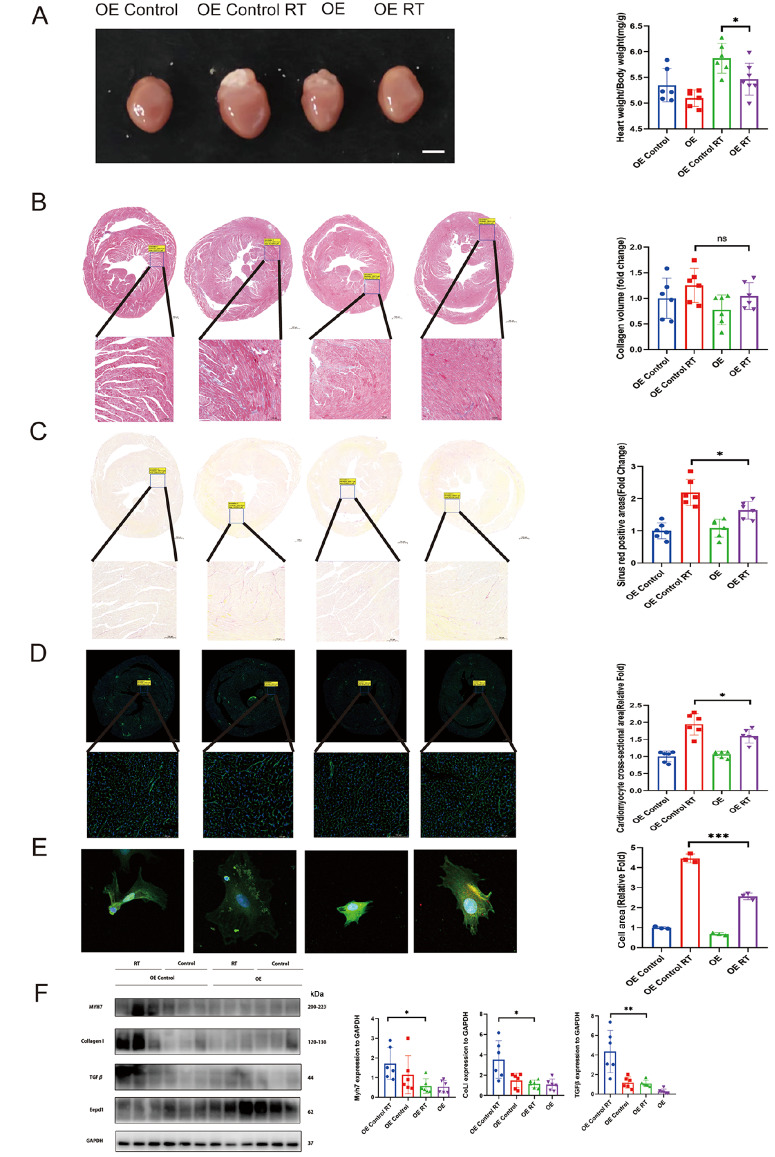
Figure 5 *EEPD1* overexpression ameliorates radiation-induced cardiac hypertrophy (A) Representative whole heart images. Scale bar: 2.5 mm. Myocardial fibrosis was detected by Masson’s trichrome staining. Scale bar: 500 μm (upper) and 100 μm (lower). (C) Myocardial fibrosis detected by Sirius Red staining. Scale bar: 500 μm (upper) and 100 μm (lower). (D) WGA staining showing myocyte hypertrophy. Scale bar: 500 μm (upper), and 100 μm (lower). (E) Immunofluorescence images of NRCMs and related quantification. Scale bar: 10 μm. (F) Representative western blots and related quantification. *P<0.05, **P<0.01, ***P<0.001.

To further verify whether *EEPD1* overexpression could ameliorate the phenotype, a rescue methodology was used. Surprisingly, the increase in size, increase in heart weight/body weight ratio, and increase in fibrosis caused by radiotherapy were significantly ameliorated by *EEPD1* overexpression, and no differences in these parameters were detected between the OE control and OE groups without radiotherapy ( Figure 5A‒D). The protein levels of Myh7 and collagen I were significantly lower in the OE *EEPD1* mice than in the WT mice after radiotherapy. There was a trend toward lower TGFβ protein level in the KO group than in the WT group, but the difference did not reach statistical significance. A slight downwards trend in Myh7, Collagen I and TGFβ expressions was observed in the OE group compared with those in the OE control group without radiotherapy ( Figure 5F). Consistent with our *in vivo* results, analysis of the cell area using α-actinin staining revealed that, compared with that in the control group, the cell area of the NRCMs in the OE-treated group significantly decreased after radiotherapy, and no difference in the cell area of the NRCMs in the OE-treated control group and OE-treated group without radiotherapy was detected ( Figure 5E). Additionally, the decreased ejection fraction and fractional shortening in the EEPD1 OE mice after radiation were alleviated. Increases in the left ventricular mass, left ventricular anterior wall, and left ventricular posterior wall were also alleviated in *EEPD1* OE mice ( Figure 2B).

### *EEPD1* overexpression alleviates radiation-induced cardiac apoptosis

**Figure 6:**
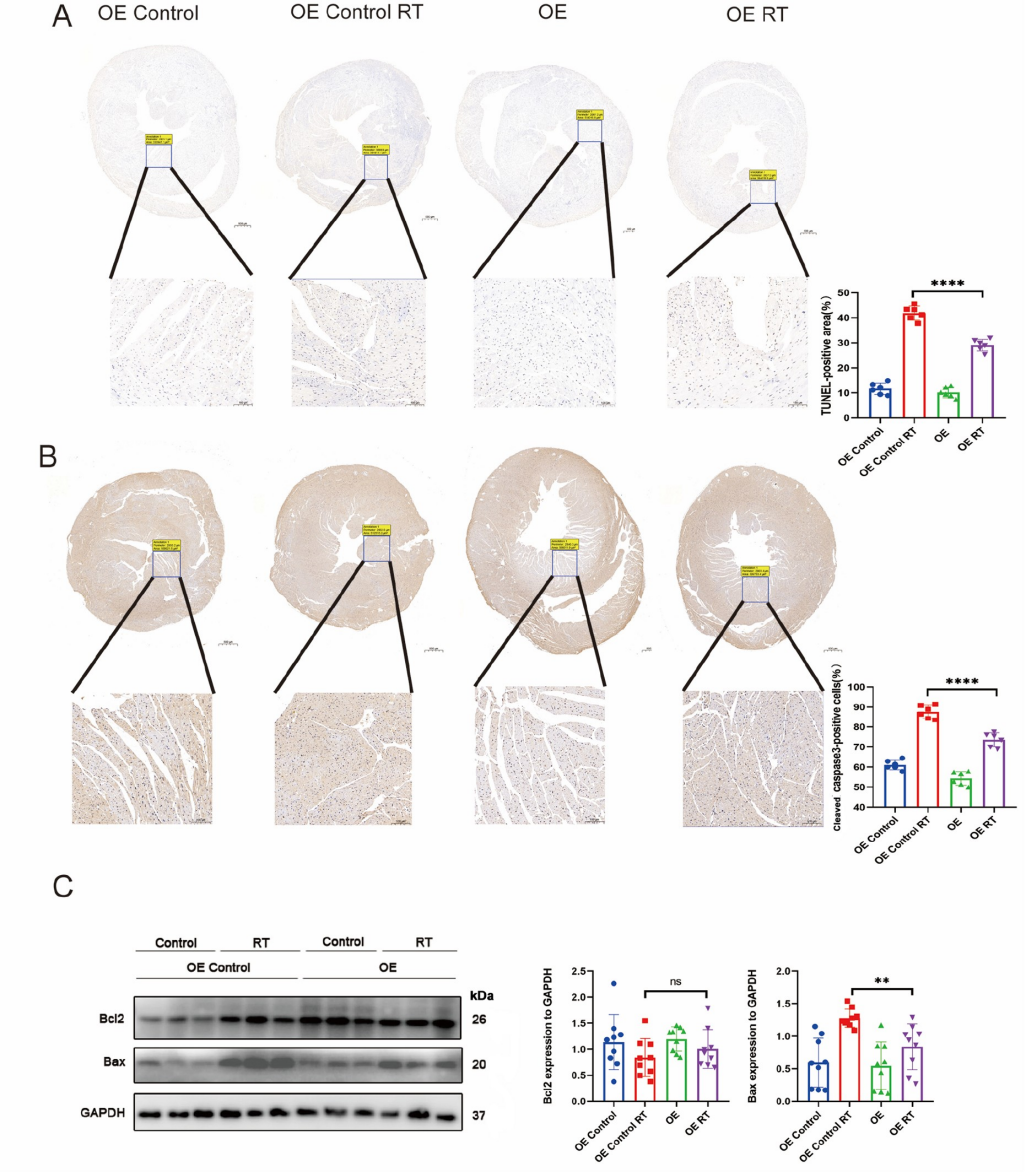
Figure 6 *EEPD1* overexpression alleviates radiation-induced cardiac apoptosis (A) TUNEL staining of each group (n=6). Scale bar: 500 μm (upper) and 100 μm (lower). (B) Representative immunohistochemical (IHC) staining of cleaved caspase-3 protein (n=6). Scale bar: 500 μm (upper) and 100 μm (lower). (C) Bcl2 and Bax protein levels in vivo. n=9 for each group. **P<0.01, ****P <0.0001.

As shown in Figure 6A,B, both TUNEL-positive and cleaved caspase-3-positive areas were significantly lower in *EEPD*1-overexpressing mice than in the WT mice after radiotherapy. No difference in the TUNEL-positive or cleaved caspase-3-positive area was observed between the OE Control and OE groups without radiotherapy. Bax protein level was significantly decreased in the OE Control mice after radiotherapy, which was ameliorated but decreased in the OE *EEPD1* mice ( Figure 6C). There was a trend toward greater Bcl2 protein level in the OE *EEPD1* group than in the OE control group, but the difference did not reach statistical significance ( Figure 6C). Furthermore, analysis of NRCMs with Annexin V-APC/7AAD staining by flow cytometry revealed that transfection with the *EEPD1* OE plasmid significantly ameliorated apoptosis after radiotherapy ( Figure 6C). These results confirmed that *EEPD1* overexpression alleviated radiation-induced cardiac apoptosis.

### *EEPD1* interacts with *FOXO3A* and destabilizes it by catalyzing its deubiquitination

**Figure 7:**
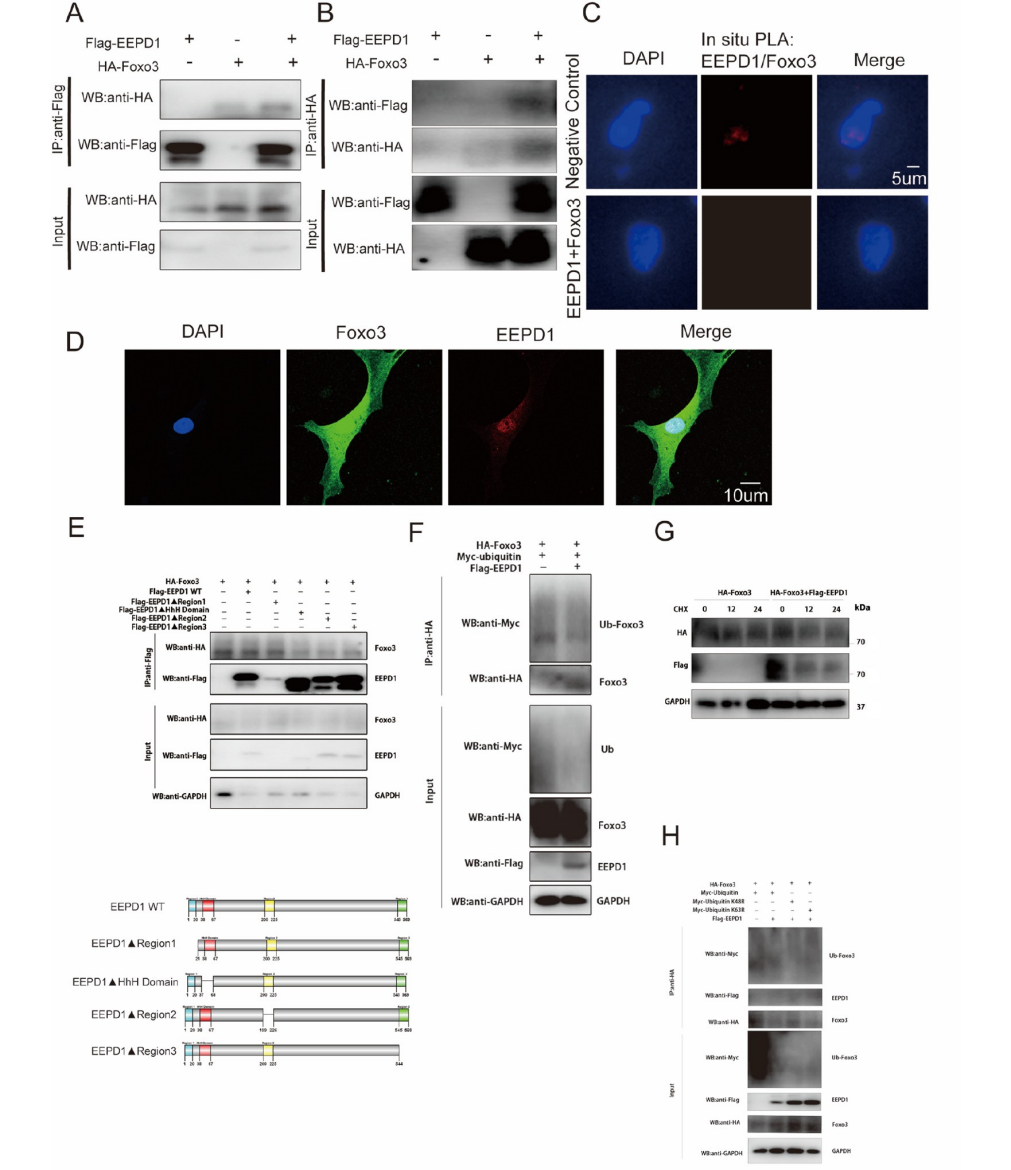
Figure 7 EEPD1 interacts with FOXO3A and destabilizes it by catalyzing polyubiquitination (A) Exogenous protein interactions were demonstrated in HEK293T cells. Lysates from HEK293T cells transfected with HA-tagged FOXO3A and Flag-tagged EEPD1 plasmids were immunoprecipitated with anti-Flag followed by western blot analysis with anti-Flag (EEPD1) and anti-HA (FOXO3A) antibodies. (B) Exogenous protein interactions were demonstrated in HEK293T cells. Lysates from HEK293T cells transfected with HA-tagged FOXO3A and Flag-tagged EEPD1 plasmids were immunoprecipitated with anti-HA, followed by western blot analysis with anti-Flag (EEPD1) and anti-HA (FOXO3A) antibodies. (C) An in situ proximity ligation assay (PLA) was performed with proximity probes against EEPD1 and FOXO3A. The nuclei were stained with DAPI (blue), and EEPD1-FOXO3A interactions were visualized with in situ PLA signals (red). Scale bar: 5 μm. (D) EEPD1 colocalized with FOXO3A in vitro. Fixed NRCMs were incubated with anti-EEPD1 and anti-FOXO3A antibodies (1/200) overnight, followed by incubation with fluorescent secondary antibodies (1/200) for 60 min. The nuclei were stained with DAPI followed by confocal fluorescence microscopy. Scale bar: 10 μm. (E) Effects of the indicated EEPD1 truncations on FOXO3A. HEK293T cells were cotransfected with HA-FOXO3A and Flag-EEPD1 constructs (ΔRegion1, ΔHhH Domain, ΔRegion2 and ΔRegion3 represent deletion mutations of the corresponding domain), and FOXO3A was analyzed. (F) Lysates from HEK293T cells transfected with Myc-tagged ubiquitin and Flag-tagged EEPD1 together with HA-tagged FOXO3A followed by treatment with MG132 for 6 h before harvest were immunoprecipitated with an anti-HA antibody, followed by western blot analysis with anti-Myc (ubiquitin) and anti-HA (FOXO3A) antibodies. (G) Representative western blot analysis of FOXO3A and EEPD1 protein expressions. HEK293T cells were transfected with Flag-EEPD1 and HA-FOXO3A or HA-FOXO3A only. The cells were then treated with cycloheximide (CHX; 10 μM) for the indicated time periods. (H) Effects of the indicated ubiquitin KR (Lys to Arg) mutants on EEPD1-mediated FOXO3A polyubiquitination. HEK293T cells were transfected with the indicated constructs, and FOXO3A ubiquitination was analyzed. The cellular extracts from HEK293T cells were pulled down with an anti-HA antibody and then analyzed by western blot analysis.

*FOXO3A* is a member of the FOXO family and responds to several cellular stresses, such as radiation and oxidative stress. A previous study suggested that activation of *FOXO3A* could alleviate pathological myocardial hypertrophy [9]. Thus, we hypothesize that *EEPD1* interacts with *FOXO3A*. In the absence of radiation, *FOXO3A* expression significantly increased in *EEPD1*-knockout mice, and this effect became more pronounced after radiation ( Figure 3F). To verify the interaction between *FOXO3A* and *EEPD1*, Co-IP was performed with Flag-tagged *EEPD1* and HA-tagged *FOXO3A* in HEK293T cells. Flag-tagged *EEPD1* coprecipitated strongly with HA-tagged *FOXO3A* ( Figure 7A). Moreover, reverse Co-IP further confirmed that HA-tagged *FOXO3A* was strongly precipitated by Flag-tagged *EEPD1* ( Figure 7B). These results suggest that *EEPD1* interacts with *FOXO3A* exogenously. Furthermore, we validated the interaction between *EEPD1* and *FOXO3A* via a proximity ligation assay in NRCMs ( Figure 7C). To explore the spatial relationship between *FOXO3A* and *EEPD1*, we performed immunofluorescence microscopy. Confocal microscopy demonstrated that *FOXO3A* and *EEPD1* colocalized in the nuclei of NRCMs ( Figure 7D). To determine whether the functional domain of *EEPD1* interacts with *FOXO3A*, we subcloned full-length *EEPD1* (FL; aa 1‒569) and various deletion mutants ( *i*. *e*., region 1 deletion (ΔRegion1; aa 1‒20]; HhH domain deletion (ΔHhH; aa 38‒67]; region 2 deletion (ΔRegion2; aa 200‒225); and region 3 deletion (ΔRegion3; aa 545‒569) mutants into flag-CMV plasmids. HEK293A cells were transfected with the HA- *FOXO3A* plasmid and Flag- *EEPD1* plasmid, which flanked the *EEPD1* FL/ΔRegion1/ΔHhH domain/ΔRegion2/ΔRegion3 plasmids, respectively ( Figure 7E), and then coimmunoprecipitation analysis was performed using an anti-HA antibody. All the *EEPD1* FL, *EEPD1*ΔRegion1 and *EEPD1*ΔRegion3 mutants were specifically immunoprecipitated by *FOXO3A* via the HA antibody. However, the *EEPD1*ΔHhH domain and the *EEPD1*ΔRegion2 mutants were not immunoprecipitated by *FOXO3A*, suggesting that the ΔHhH domain (aa 38‒67) and Region2 (aa 200‒225) mediate the *FOXO3A*– *EEPD1* interaction ( Figure 7E). Ubiquitination is an important mechanism for regulating protein stability; therefore, we studied whether *EEPD1* regulates *FOXO3A* ubiquitination and degradation. HEK293T cells were cotransfected with the HA- *FOXO3A* and Myc-ubiquitin plasmids with or without Flag-EEPD1. As expected, overexpression of *EEPD1* decreased *FOXO3A* ubiquitination ( Figure 7F). Next, we inhibited protein translation by incubating the cells with cycloheximide and determined the HA-FOXO3A protein level and stability by western blot analysis. Compared with control treatment, *EEPD1* overexpression significantly decreased FOXO3A protein level ( Figure 7G). Moreover, K48- and K63-linked chains are the two most abundant polyubiquitin chain types that regulate proteolytic and signaling pathways, respectively. We then aimed to specify which polyubiquitin chain type is mediated by *EEPD1*. HEK293T cells were transfected with Flag-EEPD1, HA-FOXO3A and Myc-ubiquitin (WT, Ub-K48R, or Ub-K63R) plasmids. Our results showed that UbK63R but not Ub-K48R ameliorated *EEPD1*-mediated *FOXO3A* deubiquitination (H Figure 7H).

### Inhibition of *FOXO3A* ameliorates cardiac hypertrophy after *EEPD1* knockdown

**Figure 8:**
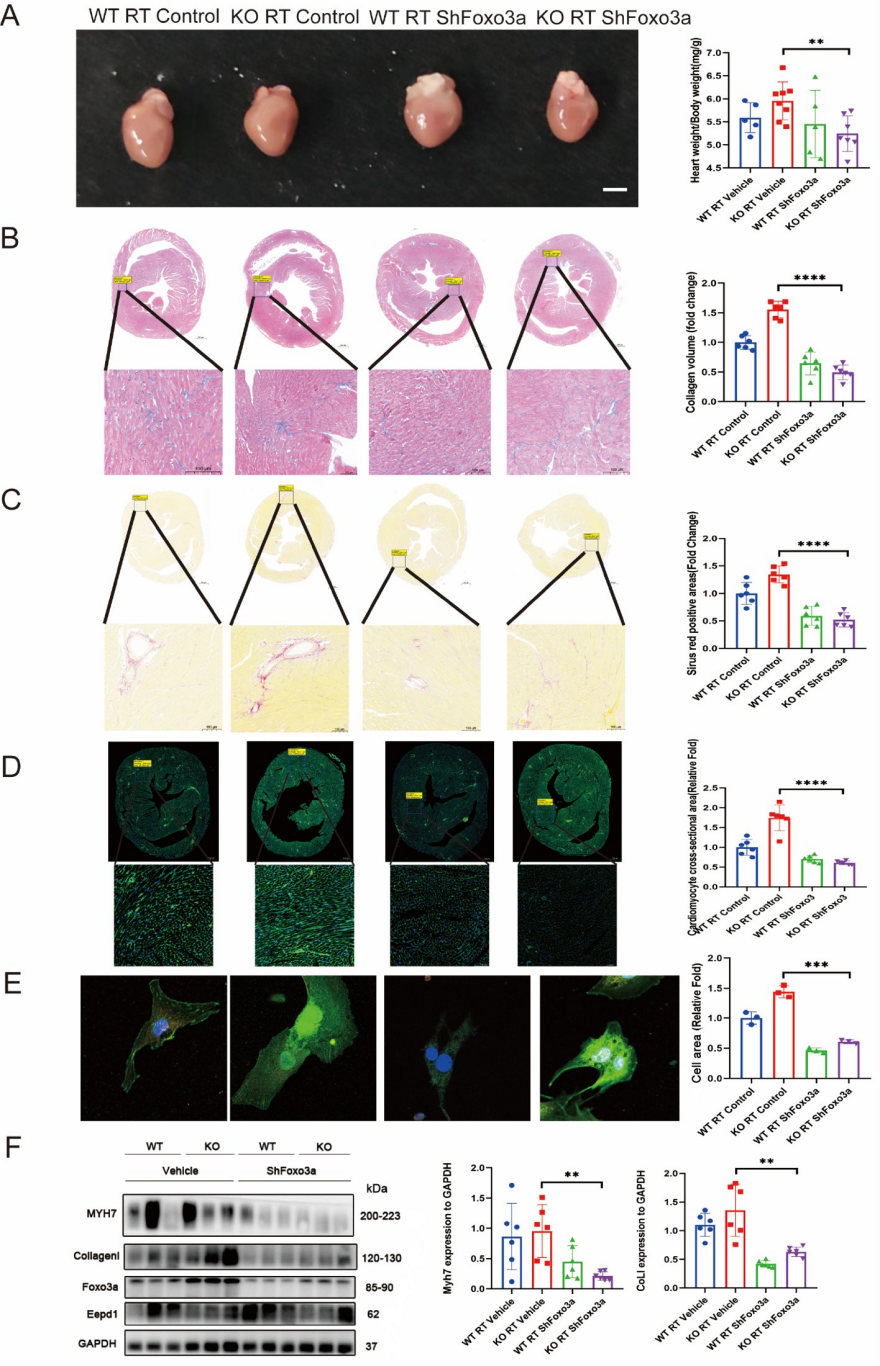
Figure 8 Inhibition of FOXO3A ameliorates cardiac hypertrophy after *EEPD1* knockdown (A) Representative whole heart images. Scale bar: 2.5 mm. Myocardial fibrosis was detected by Masson’s trichrome staining. Scale bar: 500 μm (upper) and 100 μm (lower). (C) Myocardial fibrosis detected by Sirius Red staining. Scale bar: 500 μm (upper) and 100 μm (lower). (D) WGA staining showing myocyte hypertrophy. Scale bar: 500 μm (upper), and 100 μm (lower). (E) Immunofluorescence images of NRCMs and related quantification. Scale bar: 10 μm. (F) Representative western blots and related quantification. ** P<0.01, ***P<0.001, ****P <0.0001.

To investigate whether *EEPD1* regulates radiation-induced cardiac hypertrophy through *FOXO3A*, *EEPD1*-knockdown and WT mice were injected via the tail vein with Sh *FOXO3A*. As predicted, the increase in size, increase in heart weight/body weight ratio, and increase in fibrosis caused by radiotherapy in *EEPD1-*knockdown mice were significantly ameliorated by inhibition of *FOXO3A* ( Figure 8A‒D). After radiotherapy, the protein levels of Myh7 and collagen I were significantly lower in the KO+sh *FOXO3A* mice than in the KO+shControl mice ( Figure 8F). Consistent with our *in vivo* results, analysis of the cell area using α-actinin staining revealed that the FOXO3 inhibitor significantly decreased the cell area of sh *EEPD1* NRCMs after radiotherapy compared to that in the control group ( Figure 8E). The ejection fraction and fractional shortening in *EEPD1-*knockdown mice increased after *FOXO3A* inhibition ( Supplementary Figure S3), while the left ventricular mass, left ventricular anterior wall, and left ventricular posterior wall decreased ( Supplementary Figure S3).

### Inhibition of *FOXO3A* alleviates cardiac apoptosis after *EEPD1* knockdown

**Figure 9:**
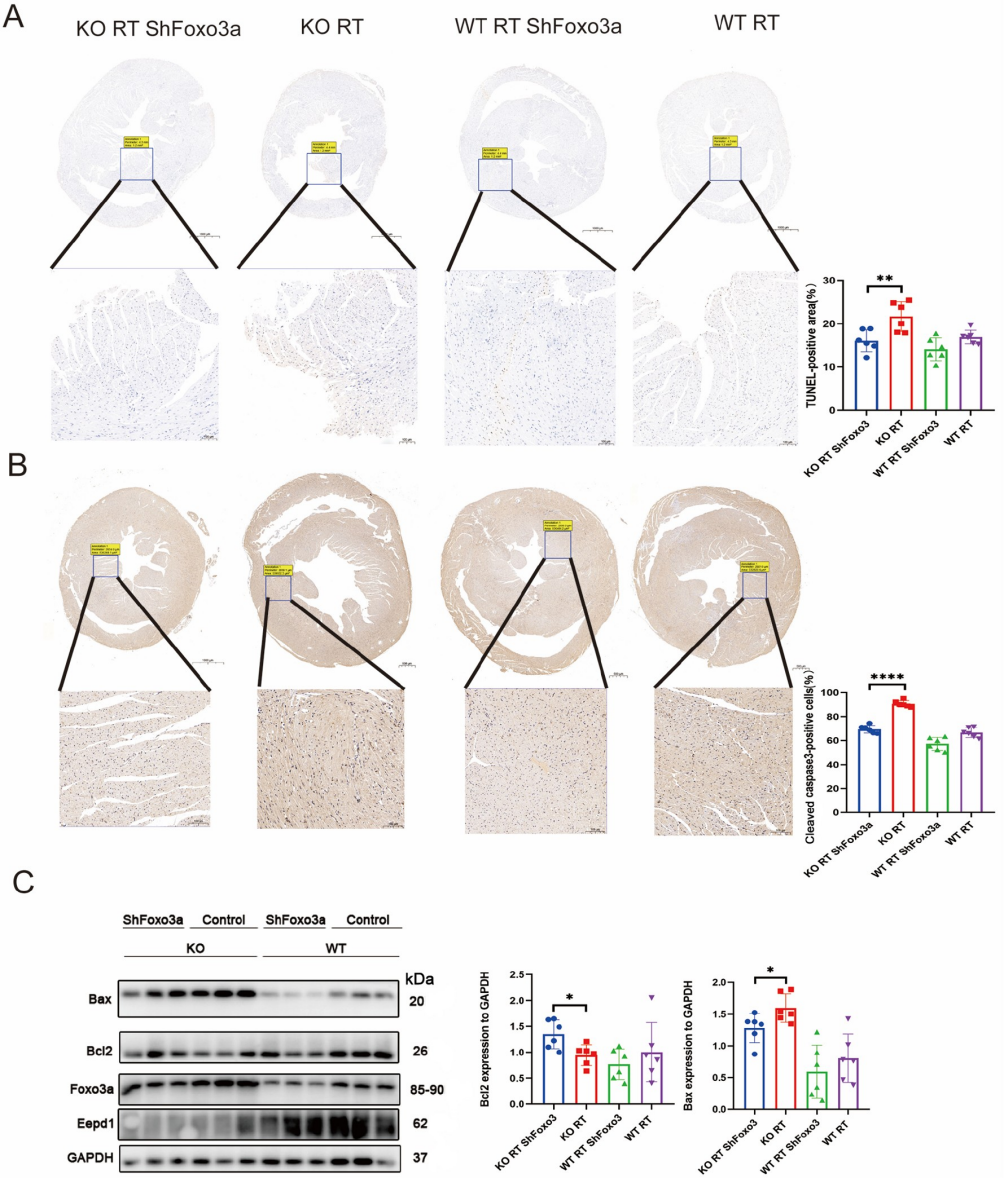
Figure 9 Inhibition of FOXO3A alleviates cardiac apoptosis after *EEPD1* knockdown (A) TUNEL staining of each group. (B) Representative immunohistochemical (IHC) staining of cleaved caspase-3 protein. (C) Bcl2, Bax, and FOXO3A protein levels in vivo . n=6 for each group. *P<0.05, **P<0.01, ****P<0.0001.

We next asked whether modulation of *FOXO3A* could elicit cardiac apoptosis after *EEPD1* knockdown. *EEPD1*-knockdown and WT mice were injected via the tail vein with Sh *FOXO3A*. As shown in Figure 9A, interference with *FOXO3A* significantly reduced the TUNEL-positive area in *EEPD-*knockout mice after radiotherapy. Consistent with this result, the cleaved caspase-3-positive area decreased after *FOXO3A* inhibition in *EEPD1*-knockdown mice ( Figure 9B). Bax protein level significantly decreased and Bcl2 level significantly increased after *FOXO3A* inhibition in *EEPD1*-knockout mice ( Figure 9C).

## Discussion

RIHD is a serious complication of radiation therapy and is difficult to manage. RIHD not only reduces quality of life but also affects treatment decision-making. At present, most studies on RIHD have focused on cardiac endothelial cells, especially the microvasculature. A large number of mechanisms, such as inflammation [ 18, 19] and mitochondrial dysfunction [ 20 , 21] are associated with cardiac endothelial cells in RIHD.

Hypertrophy initially develops as an adaptive response to physiological and pathological stimuli, but pathological hypertrophy generally progresses to heart failure [22]. Angiotensin II (Ang II) is a well-known cause of hypertension and cardiac hypertrophy. Moreover, a variety of factors can induce cardiac hypertrophy. Sun *et al*. [23] reported that PDE1C activation and ER stress contribute to the development of cardiac hypertrophy induced by homocysteine. In addition, cyclic nucleotides, especially cAMP, are downstream mediators of the ER stress-PDE1C signaling axis. Xie *et al*. [24] reported that USP28 regulates mitochondrial homeostasis via the PPARα-Mfn2 axis and modulates cardiac hypertrophy in diabetic cardiomyopathy. Yang *et al*. [25] reported that hydrogen attenuates thyroid hormone-induced cardiac hypertrophy in rats by regulating angiotensin II type 1 receptor- and NADPH oxidase 2-mediated oxidative stress. Here we showed that radiation can also induce cardiac hypertrophy.

In this study, we found that radiation markedly downregulated cardiac *EEPD1* level in the RIHD group compared to that in the control group. Physiological cardiac hypertrophy is an important compensatory mechanism in RIHD, and severe cardiac hypertrophy can ultimately develop into pathological hypertrophy and heart failure if not effectively disrupted [ 26, 27]. In addition, myocardial apoptosis is also an important factor in many heart diseases, such as septic cardiomyopathy [28], myocardial infarction [29], and RIHD [30]. This finding is in agreement with clinical observations and our findings, to a certain extent. We found that *EEPD1* deficiency exacerbated cardiac apoptosis and hypertrophy in mice after radiation challenge, while *EEPD1* overexpression ameliorated these effects. These phenomena could be explained by the potential function of *EEPD1* as a cardioprotective gene. *EEPD1* deficiency causes more severe cardiac apoptosis after radiation, leading to overcompensation of the viable myocardium and ultimately causing viable myocardial apoptosis. Thus, a vicious cycle is formed.

The *EEPD1* gene belongs to the ribonuclease gene family. It has been shown to play an important role in the DNA damage repair system [ 5, 31]. *FOXO3A*, a member of the FOXO subfamily, is related to a variety of cellular processes, including apoptosis [32], proliferation [33], cell cycle progression and DNA damage [34]. A large number of studies have indicated that *FOXO3A* plays an important role in cardiac hypertrophy [ 9, 10, 35] . *FOXO3A* may affect cardiac hypertrophy by regulating the phosphorylated glycogen synthase kinase-3β (p-GSK3β)/β-catenin/cyclin D1 signaling pathway, the ratio of p- *FOXO3A* to *FOXO3A*, and the nuclear level of *FOXO3A*. Because of the functional similarity and significant increase in *FOXO3A* after *EEPD1* knockout in RIHD, we hypothesized that *FOXO3A* may be a downstream protein of *EEPD1*. Here, the interaction between *FOXO3A* and *EEPD1* was confirmed by co-localization, Co-IP and proximity ligation assays. We also found that *EEPD1* destabilized *FOXO3A* through K63-linked polyubiquitination. The ΔHhH domain (aa 38‒67) and ΔRegion2 domain (aa 200‒225) of *EEPD1* interact with *FOXO3A*. *FOXO3A* contains five domains: a highly conserved forkhead winged helix-turn-helix DNA binding domain (FKH), two nuclear localization sequences (NLSs), a nuclear export sequence (NES) and a C-terminal transactivation domain (TAD). However, which domain interacts with *EEPD1* was not determined in our study due to time constraints. The inhibition of *FOXO3A* ameliorated cardiac apoptosis and hypertrophy caused by *EEPD1* knockdown in RIHD, whichwas in accordance with the findings of a previous study [ 32, 36]. We hypothesize that in RIHD, the *EEPD1*-Foxo3 axis may ultimately lead to mitochondrial depolarization and apoptosis by regulating BNIP3 [37].

There were several limitations in this study: 1) the absence of conditional *EEPD1* knockout mice and 2) the need for further investigation into the upstream molecular mechanism involved.

In summary, our findings provide novel evidence supporting the protective role of myocardial *EEPD1* in RIHD. This study demonstrated that radiation downregulated myocardial *EEPD1*, leading to the upregulation of *FOXO3A* and subsequent exacerbation of apoptosis and cardiac hypertrophy. These effects were ameliorated by *FOXO3A* inhibition and destabilization by *EEPD1* through deubiquitination. The *EEPD1*- *FOXO3A* pathway may serve as a potential therapeutic target for RIHD, and future selective targeting of myocardial *EEPD1* to reduce the activation of *FOXO3A* should have a localized effect on RIHD without significantly influencing systemic innate immune responses.

## Supplementary Data

Supplementary data is available at *Acta Biochimica et Biophysica Sinica* online.

## Supporting information

23608supplementary_Figures
