# Supplementary material for: EEPD1 attenuates radiation-induced cardiac hypertrophy and apoptosis by degrading FOXO3A in cardiomyocytes : Effect of EEPD1 on radiation induced cardiomyopathy
Source: Acta Biochim Biophys Sin (Shanghai). 2024 Aug 29;56(12):1733–47. doi: 10.3724/abbs.2024130 (PMC11659772; doi:10.3724/abbs.2024130)
Supplement: 23608supplementary_Figures [file 23608supplementary_Figures.pdf]

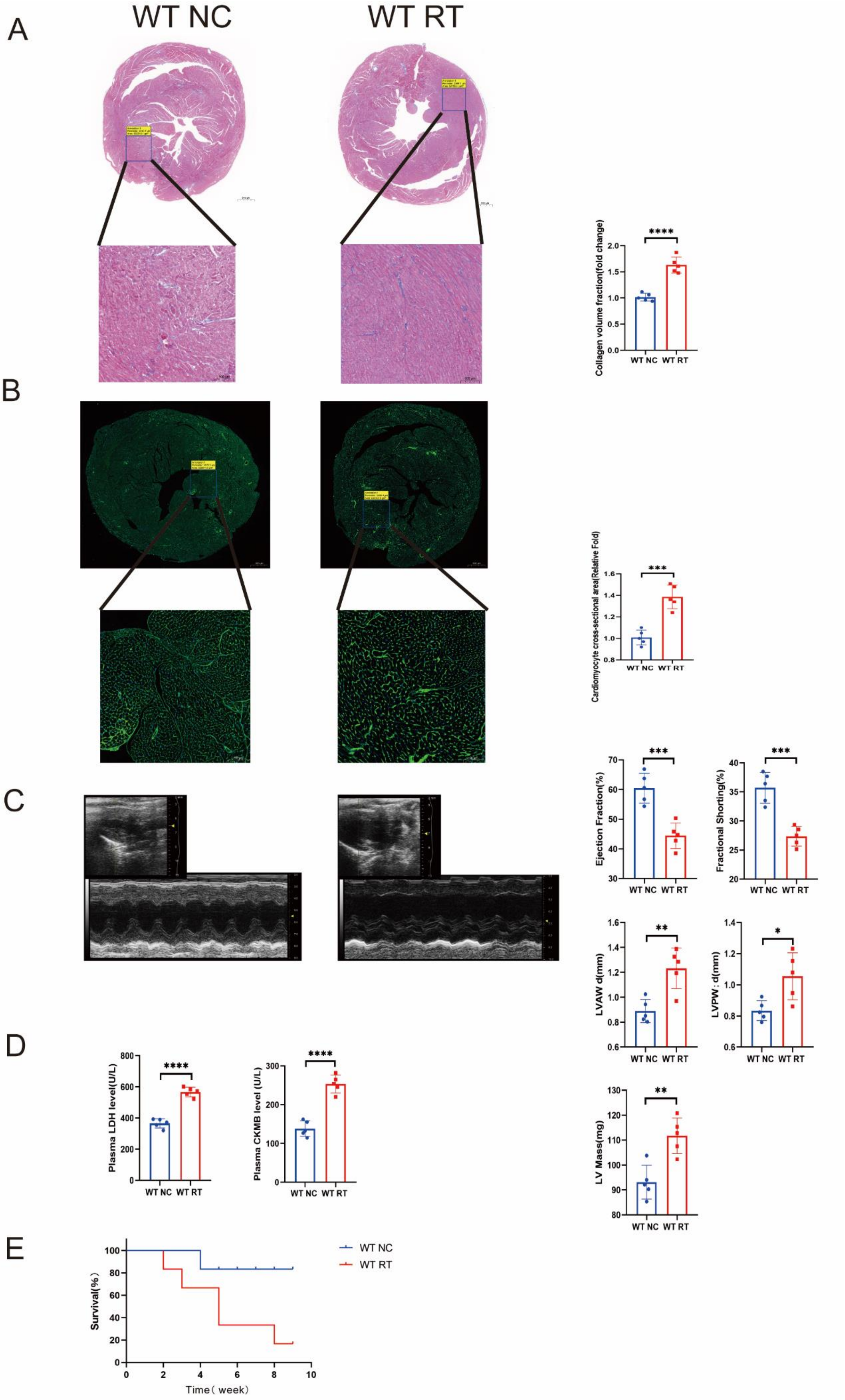
A
WT NC
WT RT
Collagen volume fraction(fold change)
****
2.0
1.5
1.0
0.5
0.0
WT NC
WT RT
B
Cardiomyocyte cross-sectional area(Relative Fold)
***
1.6
1.4
1.2
1.0
0.8
WT NC
WT RT
C
Ejection Fraction(%)
***
70
60
50
40
30
WT NC
WT RT
Fractional Shorting(%)
***
40
35
30
25
20
WT NC
WT RT
LVAW d(mm)
**
1.6
1.4
1.2
1.0
0.8
0.6
WT NC
WT RT
LVPW; d(mm)
*
1.4
1.2
1.0
0.8
0.6
WT NC
WT RT
D
Plasma LDH level(U/L)
****
800
600
400
200
0
WT NC
WT RT
Plasma CKMB level (U/L)
****
300
200
100
0
WT NC
WT RT
LV Mass(mg)
**
130
120
110
100
90
80
WT NC
WT RT
E
Survival(%)
100
80
60
40
20
0
0
2
4
6
8
10
Time (week)
WT NC
WT RT

Supplementary Figure S1. the success of the model by Masson staining, WGA staining, echocardiography, cardiac enzyme analysis, and an altered survival rate (A) Myocardial fibrosis detected by Masson's trichrome staining. Scale bars: 500 μm (upper) and 100 μm (lower). (B) Wheat germ agglutinin (WGA) staining of myocyte hypertrophy. Scale bars: 500 μm (upper), and 100 μm (lower). (C) Representative images of echocardiographs and statistics of ejection fraction (EF), fractional shortening (FS), LV Mass, LVAW d and LVPW. (D) Effect of radiation on CKMB and LDH release. (E) Survival rate analysis.

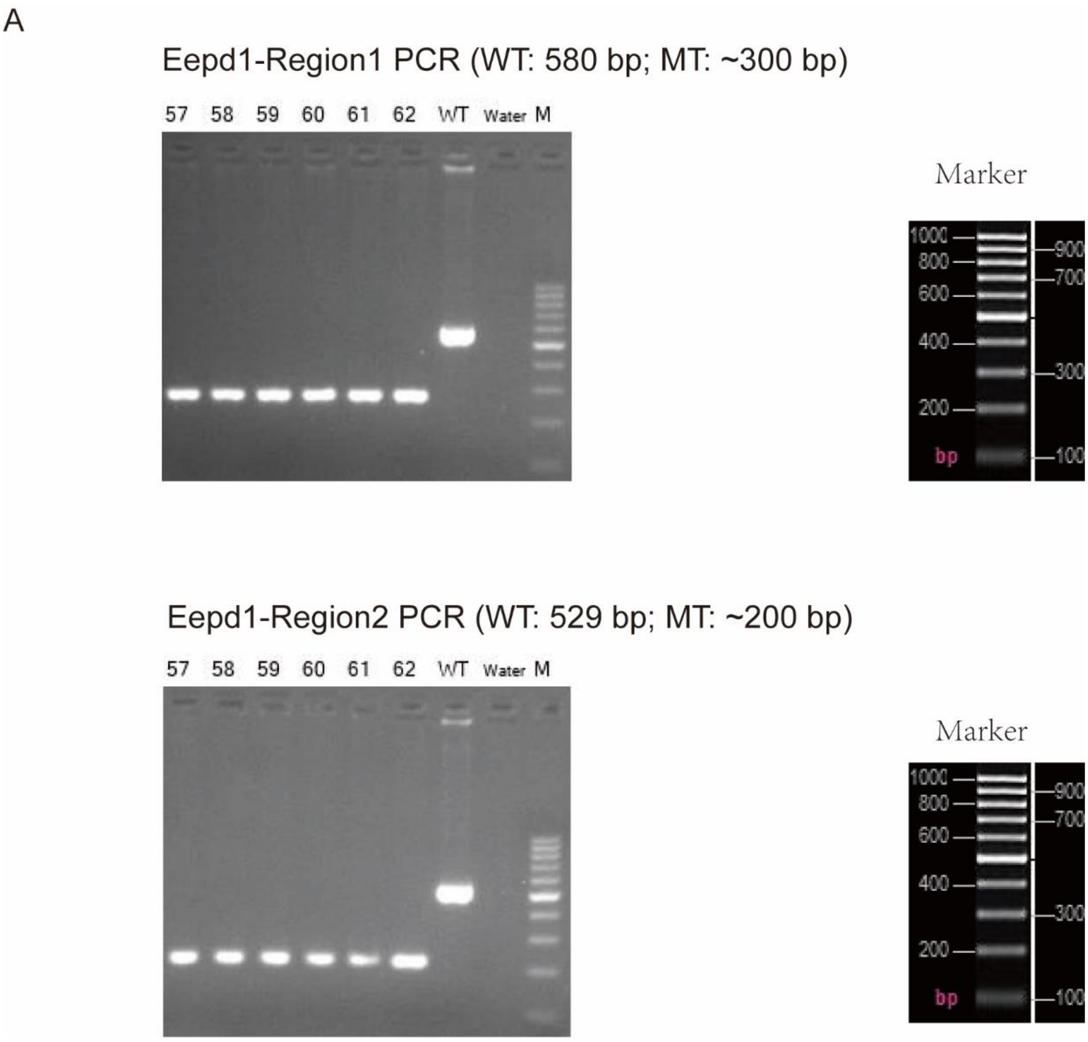

Supplementary Figure S2 PCR analysis (A) Six pups (57#, 58#, 59#, 60#, 61# and 62#) were identified homozygotes by PCR screening for Eepd1-Region1. Six pups (57#, 58#, 59#, 60#, 61# and 62#) were identified homozygotes by PCR screening for Eepd1-Region2. (B) Six pups (57#, 58#, 59#, 60#, 61# and 62#) were identified homozygotes by PCR screening for Eepd1-Region1 and Eepd1-Region2.

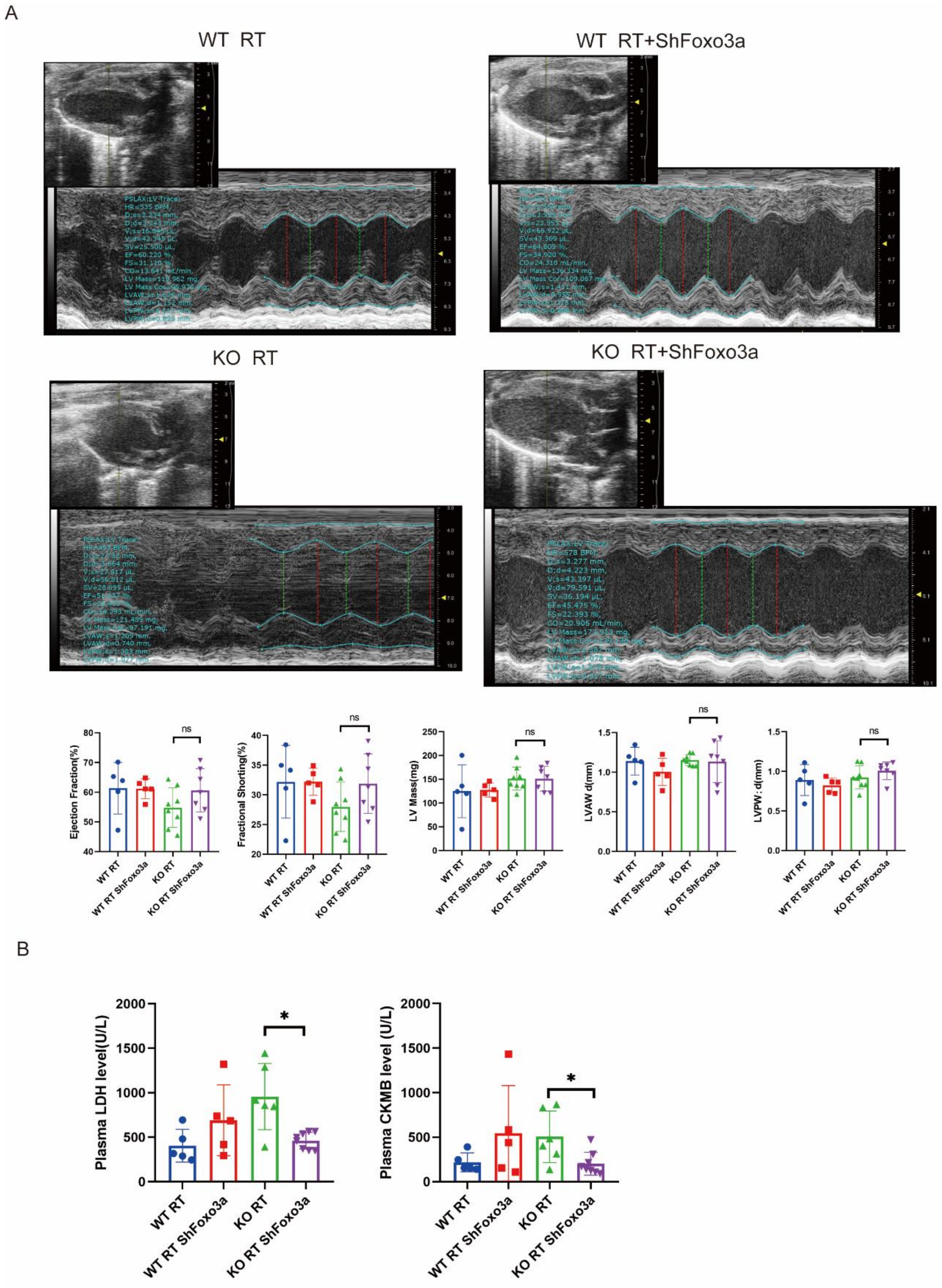

Supplementary Figure S3 (A) Representative images of echocardiographs and statistics of ejection fraction (EF), fractional shortening (FS), LV Mass, LVAW d and LVPW d. (B) Effect of CKMB and LDH release after radiotherapy.
